# Editorial: The functional connectome of chemosensory perception

**DOI:** 10.3389/fnsys.2022.1025962

**Published:** 2022-10-12

**Authors:** Florian Ph.S Fischmeister, Maria G. Veldhuizen, Cinzia Cecchetto

**Affiliations:** ^1^Institute of Psychology, University of Graz, Graz, Austria; ^2^Department of Biomedical Imaging and Image-Guided Therapy, Medical University of Vienna, Vienna, Austria; ^3^BioTechMed-Graz, Graz, Austria; ^4^Department of Anatomy, Faculty of Medicine, Mersin University, Mersin, Turkey; ^5^Department of General Psychology, University of Padova, Padua, Italy

**Keywords:** chemosensory perception, editorial, connectome, human, olfaction, taste, chemesthesis

Sensory stimuli allow us to generate a vivid representation of our environment, which in turn forms the basis for our behavior and provokes cognition. However, sensory stimuli rarely trigger neuronal areas in isolation but activate a whole network of brain regions interacting across time and space. This is particularly true for ecological, chemosensory stimuli consisting of three heterogeneous sensory modalities: smell, taste, and chemesthesis. Since such stimuli commonly evoke more than one sensory modality simultaneously, the integration into a unitary percept has to be achieved by interactions between multiple brain areas. Such a multisensory percept is further enriched by information from other sensory modalities, memory, or higher cognition. In turn, chemosensory perceptions play a crucial role in regulating emotions and modulating social interaction, nutrition, and well-being. Therefore, understanding these central neuronal interactions is a par excellence necessity for understanding multimodal chemosensory perception.

With the advent of network neuroscience, there is an ever-growing body of research and methods studying brain organization, aiming to describe connections and their properties between different elements of our nervous system. This development is primarily driven by the advances in complex network theory leading to the new field of connectomics, initially described as “...a comprehensive structural description of the network of elements and connections forming the human brain.” by Sporns et al. (2005).

So while there has been tremendous progress in the analysis of the interaction between brain regions, enhancing our understanding of the structural and functional mechanism underlying brain network organization, the field of chemosensory neuroimaging is still dominated by studies examining the role and contribution of single brain regions (see Veldhuizen et al.). Nevertheless, throughout recent years, our understanding of peripheral and central mechanisms underlying our ability to sense the chemical environment increased and led to a good understanding of the specific and distinct neuronal systems associated with each of the three chemical senses separately. However, to completely understand the neural processing of chemosensation, we must also examine interactions between active brain regions to gain a more profound knowledge of their network organization to explain and predict how chemosensory processing shapes cognition and behavior.

This Research Topic explores the functional connectome of multimodal chemosensory perception and its interconnection to other sensory and cognitive systems at various scales across species. Foremost, Farruggia et al. conducted a literature review summarizing functional connectivity papers related to olfaction, gustation, and chemesthesis. The 103 articles discovered in total showed that regardless of modality, most studies primarily focus on correlates of stimulus qualities such as identity, pleasantness, and intensity with task-based paradigms. As noted by the authors, this circumstance leads to a relative lack of paradigm-free resting-state literature, which would allow for a better understanding of chemosensory networks without being influenced by motion artifacts commonly seen with gustatory or olfactory paradigms (see also Veldhuizen et al. in this issue). Fjaeldstad et al. then aim to validate the functional olfactory connectome from a structural perspective. To this end, they combine diffusion data with an anatomical atlas and use advanced probabilistic tractography to show a robust structural connectivity pattern between primary olfactory cortices and key secondary olfactory regions. Furthermore, they show that this structural network pattern potentially encoded individual differences in olfactory behavior.

Aiming in a different direction but still within the domain of olfaction, Noto et al. investigate subregions of the amygdala receiving projections from the olfactory bulb. Following a literature review on three amygdala subregions, they use resting-state data to show that the anatomical boundaries of these subregions can be parcellated accurately. This finding suggests that the associated functional networks are distinct from other amygdala subregions not receiving input from the olfactory bulb. Based on these analyses, the authors finally speculate on the functions of these regions with respect to olfaction and suggest further investigating them as part of the primary olfactory cortex.

In a cross-modal study, Invitto et al. then used EEG to investigate the putative role of pheromones or social odors and gender voice in humans and how they affect the behavioral and psychophysiological states. Based on behavioral data, Invitto et al. conclude that these social odors do influence measures of co-presence and social presence gender specifically. Employing transfer entropy analysis of acquired EEG data, the authors could then replicate and expand these findings showing corresponding differences in right frontotemporal regions (involved in odor recognition memory and social behavior) and the bilateral frontoparietal network.

Moving from olfaction to the taste domain, Hartig et al. assessed the laterality, modularity, and centrality of networks involved in processing sweet, salty, and sour tastes in anesthetized macaque monkeys. Using a data-driven approach, they observe three bilateral sub-networks that reflect the salience and interoception processing networks through a medial-lateral-subcortical organization. They also note that the networks resemble each other in the differential processing of taste qualities, with more robust responses observed for sweet tastes and stronger network connectivity for sour and salty tastes. In their review of the rodent literature, Samuelsen and Vincis argue for an intraoral cortical hub in which the piriform, gustatory, and somatosensory cortical regions interact to produce the complex sensation of flavor and the ensuing consummatory behavior. Furthermore, they argue for the field to embrace the multifaceted nature of foods and beverages in their study design. From a different perspective, Nakamura and Koike used palatable liquid consumption fMRI and resting-state fMRI to explore the association between disinhibited eating and trait impulsivity to eating behavior in healthy adolescents. They found that increased disinhibited eating and increased impulsivity were positively associated with greater insular and greater amygdala responses to palatable liquid consumption. The functional connectivity analyses also showed that increased disinhibited eating was associated with enhanced intrinsic functional connectivity between the insula and the amygdala. Since both amygdala and insular cortex are involved in trait impulsivity and eating behavior, the authors suggested that heightened disinhibited eating, in combination with impulsivity, modulates neural reactivity to palatable flavors driving adolescents to maladaptive eating.

Finally, Veldhuizen et al. propose future directions for investigating the chemosensory connectomes and describe best practices and specific challenges. In this collaborative manuscript by the editors and many contributors of this special issue, we first highlight some recent work in chemosensory connectomics and then describe and summarize different connectomics techniques. In the second part of the manuscript, we outline specific challenges for chemosensory connectome neuroimaging studies and conclude with best practices from the general connectomics and neuroimaging fields.

The field of chemosensory connectomics is undoubtedly still in its infancy, and much work is required till it reaches today's majority of, e.g., the visual connectome. Throughout the preparations and while working as editors for this Research Topic, it became apparent that there are many and sometimes very different approaches to describe aspects of the chemosensory connectome. However, among other things, a complete description of the chemosensory connectome across all three modalities is desperately missing. Without such a thorough description of the connectome, the beauty of chemosensory perception, its possibilities, and its influence on our daily lives will always be only partially solved.

## Author contributions

All authors wrote parts of this editorial and contributed to manuscript revision, read, and approved the submitted version.

## Funding

MV was supported by the 2232 International Fellowship for Outstanding Researchers Program of TÜB ITAK under award number 118C299. CC was supported by a grant of the Department of General Psychology from MIUR (Dipartimenti di Eccellenza DM May 11, 2017 n. 262). FF was supported by the FWF, Austrian Science Fund, through grant KLI-639 and I-418.

## Conflict of interest

The authors declare that the research was conducted in the absence of any commercial or financial relationships that could be construed as a potential conflict of interest.

## Publisher's note

All claims expressed in this article are solely those of the authors and do not necessarily represent those of their affiliated organizations, or those of the publisher, the editors and the reviewers. Any product that may be evaluated in this article, or claim that may be made by its manufacturer, is not guaranteed or endorsed by the publisher.
